# Molecular Targets of 20-Hydroxyecdysone in Mammals, Mechanism of Action: Is It a Calorie Restriction Mimetic and Anti-Aging Compound?

**DOI:** 10.3390/cells14060431

**Published:** 2025-03-13

**Authors:** Ernő Zádor

**Affiliations:** Institute of Biochemistry, Albert Szent-Györgyi Medical School, University of Szeged, 6720 Szeged, Hungary; zador.erno@med.u-szeged.hu

**Keywords:** 20-hydroxyecdysone, 20E, ecdysterone, beta-ecdysone

## Abstract

The 20-hydroxyecdysone (20E) has been used in traditional medicine for a long time and acquired attention in the last decade as a food supplement and stimulant in physical activities. This polyhydroxylated cholesterol is found in the highest concentration in plants, and it is one of the secondary plant products that has a real hormonal influence in arthropods. Various beneficial effects have been reported in vivo and in vitro for 20E and its related compounds in mammals. Trials for the safety of clinical application showed a remarkably high tolerance in humans. This review aims to assess the latest development in the involvement of various pathways in tissues and organs and look if it is plausible to find a single primary target of this compound. The similarities with agents mimicking calorie restriction and anti-aging effects are also elucidated and discussed.

## 1. Introduction

**Figure 1:**
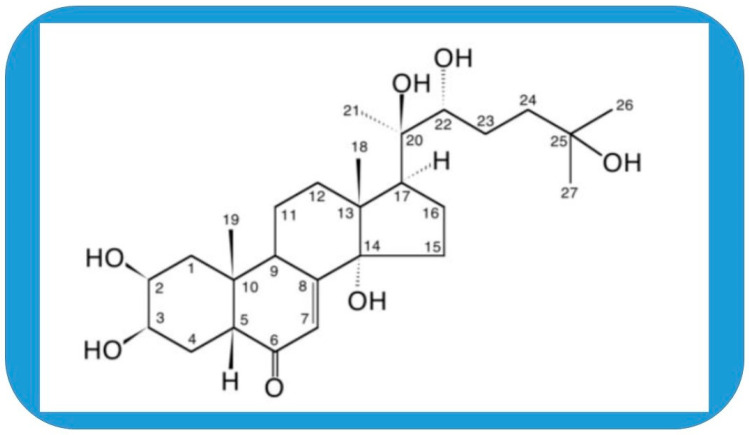
The structure of 20-hydroxyecdysone (20E, ecdysterone, β-ecdysterone, β-ecdysone, moulting hormone, and BIO101).

Phytoecdysteroids have been found to exert mostly beneficial effects on mammals. In fact, hardly any detrimental results have been observed after the administration of these compounds [1,2,3]. This is in contrast to steroid hormones that are endogenous in mammals that usually accompany the positive anabolic effect with an often dangerous androgenic one [4,5,6]. Phytoecdysteroids seem to be pure anabolic, and many plants that contain them are traditionally used in phytomedicine [1,7,8]. This property has generated remarkable attention in the past decades from not only the side of natural compound research but also sport and medical science. In vitro experiments have identified several molecular targets for the most investigated phytoecdysteroid, the 20-hydroxyecdysone (20E, beta-ecdysterone, ecdysterone, beta-ecdysone, and moulting hormone; Figure 1). First, 20E-induced hypertrophy and protein synthesis were prevented by GPCR inhibitor in differentiating C2C12 myoblasts [9,10]. The hypertrophying effect of 20E on these cells was similar to that of the beta-oestrogen and was prevented by the beta-oestrogen receptor-selective antagonist. However, no direct binding of 20E has been demonstrated to the beta-oestrogen receptor [11], and involvement of a membrane-bound receptor was supported by the effectiveness of a protein-bound 20E [12]. Interestingly, angiotensin 1–7, the endogenous ligand of the MAS receptor, had a similar effect on hypertrophy of C2C12 myogenic cells as 20E did. The endogenous muscle growth inhibitor myostatin was partially declined by 20E or angiotensin 1–7. The effects of both ligands were abolished by a specific antagonist of angiotensin 1–7. MAS receptor, as the protective arm of the renin–angiotensin system (RAS), appeared feasible to interpret pleiotropy; therefore, a cooperative activity between MAS and a palmitoylated (membrane-bound) estrogenic receptor has been proposed for the mechanism of 20E action in a RAAS—renin–angiotensin–aldosterone system [1,12]. Recently, it has been shown that 20E exerts its effect through SIRT6-mediated deacetylation of NF-κB p65 (nuclear factor kappa of B cells) to inhibit CD40 expression in 3-D human endothelial cell culture (HUVEC). Therefore, the authors proposed that 20E may have therapeutic potential for the treatment of cardiovascular diseases [13]. In animal studies, 20E revealed anabolic, anti-oxidant, antidiabetic, anti-obesity, cardioprotective, neuroprotective, hepatoprotective, and other properties. It seems so that the multiple effects can be better explained by more than just one molecular target. In fact, as research expands, the pleiotropy of action and the complexity of the mechanism appear to increase. Highlighting overlaps and sometimes opposites of these in vitro and in vivo mechanisms in the different tissues, organs, and conditions is the main topic of this review. References have been collected from PubMed and Google. A number of citations revealed that 20E has anti-obesity effect and many similarities in the influenced molecular pathways and alleviated pathological conditions with a circle of compounds proposed for mimicking calorie restriction (CRM) and seeking an anti-aging effect. The discussion of these commonalities is where this overview aims to conclude.

## 2. Cell, Tissue, and Systematic Action

### 2.1. Effects on Skeletal Muscle

The actions of ecdysteroids on skeletal muscle were among the first ones reported (reviewed in [4]). About 16 years ago, an in vitro cellular assay was developed to investigate the effects on protein synthesis. This method showed that murine C2C12 myotubes and human primary myotubes elevated protein synthesis by up to 20% when treated with 20E. This was supported by an increase in grip strength of mice after in vivo administration of 20E. The same effect was found when ecdysteroid-containing plant extract was used. According to the canonical regulation of protein synthesis, phosphoinositide-3-kinase (PI3K) inhibitor prevented these stimulatory effects [14].

The same laboratory [9] showed that 20E elicited a rapid elevation in the cytoplasmic Ca^2+^ followed by a sustained activation of Akt and increased protein synthesis in C2C12 cells. This effect was prevented by inhibitors of G-protein-coupled receptor (GPCR), phospholipase C (PLC), and PI3K, supporting the involvement of the pathway inducing Ca^2+^ efflux from the sarcoplasmic reticulum. Besides increasing muscle mass and protein synthesis in male rats, 20E also induced IGF-I and decreased corticosterone and 17β-oestradiol levels. However, in differentiated C2C12 myoblastoma cells, each of the above hormones induced hypertrophy when administered. The hypertrophy by 20E could be reverted with anti-oestrogen but not antiandrogen. Beta-oestrogen receptor (ERβ) selective ligands also induced hypertrophy in C2C12 myotubes. Selective ligands of oestrogen receptor β but not α could prevent the 20E-induced hypertrophy in C2C12 myotubes. This suggested the involvement of ERβ in 20E action in those myotubes. However, 20E binding to ERβ was not found [11].

Muscle atrophy was alleviated by 20E in the soleus in the tenotomized leg of adult male rats. After cutting the Achilles tendon, the level of high-molecular-weight ubiquitin conjugates was decreased in the soleus but not in the plantaris muscle, suggesting attenuation of proteolysis depending on muscle type [15]. In contrast, a limited, if any, effect was found when 20E was fed to rats, and a pool of skeletal muscles had been analysed for protein kinase B/Akt level as a target of rapamycin. This result might be due to the low bioavailability of 20E in those conditions [16] or/and the outbalancing effect of muscle-specific differences. Indeed, 20E, or even its derivative, poststerone, showed muscle-specific effects when injected perimuscularly into the leg of young adult male rats [17,18]. In addition, fibre size (measured by cross-sectional area, CSA) increased depending on the fibre type. The CSA increase in different fibre types was not the same in extensor digitorum longus (EDL) as in the soleus muscles [17]. The CSA increase in fibre types also depended on the distance from application (i.e., whether the muscle was in a treated or untreated leg) and the presence of a regenerating soleus in the animal. All of these suggested strong systemic components of the effects. Moreover, 20E also increased myonuclear number in normal and regenerating muscles and augmented muscle regeneration, suggesting that it also stimulates muscle stem cells, the satellite cell activity.

Bodybuilders and various athletes have used 20E as an anabolic stimulant, although scientific data based on human trials were rarely available at that time. Such an aspect is essential for doping control; therefore, a 10-week resistant training with young volunteers, who received various doses of dietary supplements with ecdysterone, has been carried out. A significant increase in muscle size was observed in individuals who consumed the 20E-containing supplement compared to the placebo group, and, what is even more relevant to sport skills, the one-repetition bench press performance was increased. No biomarkers of liver or kidney toxicity were detected in the blood and urine samples. Although the supplement also contained leucine 4-fold below the reported effective anabolic dose, 20E has been proposed as an “anabolic agent” for the list of substances forbidden to use in sport [19]. Indeed, ecdysterone was placed in the Monitoring Program of World Anti-Doping Agency in 2020 [20].

Following this issue, the research on ecdysterone’s effect became even more relevant. Eccentric exercise is often used in training and rehabilitation or in therapy against muscle atrophy. Increased repetitions of eccentric contraction may cause muscle damage, which regenerates relatively quickly in young age. However, even small muscle damages should be avoided in conditions with old age and therapy because the muscle recovery is slower and less complete. In addition, 20E supplementation accelerated recovery of muscle integrity and function after eccentric exercise in mice. The authors suggested that this reveals potential in physical therapy and warrants further investigation [21].

As the 20E therapy looked promising against sarcopenia and atrophy, it was interesting to test if it affects aging mice. The extract of the *Ajuga turkestanica* plant, enriched in ecdysterone and pure 20E, was tested for 28 days of feeding in sedentary 20 months old male C57BL/6 mice. However, the muscle mass, fibre type and size, and the activity of the PI3K-Akt signalling pathway, an indicator of protein synthesis, were not changed, and the mRNA levels of MAFbx, MuRF-1, and myostatin were not altered in the triceps brachii and plantaris muscles of aging mice [22]. It is worth mentioning that, contrary to this study, non-aging adult mice of the same strain responded with muscle accretion in triceps brachii when 20E was administered to infusion in a previous study [23].

In spite of the extensive research, the mechanism of action and the primary molecular target of 20E still remained dubious. In C2C12 cells, the decline in myostatin, a negative regulator of muscle growth and differentiation, has been used as a reporter of anabolic activity. As the protein-bound form, 20E was still effective; the involvement of a membrane-bound receptor was assumed rather than a cytoplasmic one [12]. Angiotensin 1–7, the endogenous ligand of the MAS receptor, showed a 20E-like effect, that is, also decreased myostatin levels and increased myoblast proliferation and myotube formation. Silencing the MAS receptor with siRNA and a pharmacological inhibitor reverted the effect of 20E on myostatin expression. 17β-oestradiol (E2) also declined myostatin gene expression, but the protein-bound hormone was inactive, and its activity was not abolished by angiotensin (1–7) antagonists. Therefore, it has been proposed that 20E acts through a membrane-bound receptor, while E2 acts through a receptor that is rather cytosolic. The two receptors/pathways, the 20E action mediating MAS receptor and the membrane-bound palmitoylated oestrogen receptor, cooperate with each other in RAAS, resulting in the anabolic effect of 20E. The activation of the MAS receptor by a steroid molecule is consistent with the pleiotropic effect of ecdysterone, and it gives a feasible explanation for the similarity in angiotensin 1–7 and 20E effects [12].

The pharmaceutical grade of 20E, named BIO101, has been tested in C2C12 cells and in a sarcopenic mouse model in adult (3 mo) and old (22 mo) C57Bl6/J mice. BIO101 increased Akt/mTOR activity, the transcript levels of myogenic regulatory factors (MyoD, myogenin), and the differentiation rate of C2C12 cells (estimated by fusion index, myonuclear number, and myotube size). This effect was similar to that received from angiotensin (1–7) and was prevented by a specific antagonist of the MAS receptor. Chronic oral treatment of C57Bl6/J mice (4 weeks of adults and 14 weeks of old ones) with BIO101 (50 mg/kg/day) elevated Akt/mTOR in gastrocnemius and increased muscle size, running distance, and velocity performance [24]. Since the same dose of 20E has been used as in [22], the anabolic effect and increased endurance might be due to the different mouse strain used and the longer treatment (14 vs. 4 weeks) applied. Another interesting result of this study was that old mice on a high-fat diet were reluctant to gain weight, but this can be improved with BIO101 [24]. This observation strengthened the long-standing experience that 20E can be used in not only sports nutrition but also health therapies [25]. It is also consistent with the effect of 20E on the MAS receptor. By this receptor, a non-classical renin–angiotensin (RAS) pathway is mediated that mitigates the classical RAS pathway operated by angiotensin II and angiotensin type I receptors acting for body weight loss by increasing ROS [26]. As it has been proposed, the 20E-stimulated MAS receptor may cooperate with a palmitoylated oestradiol receptor in C2C12 cells and increase protein synthesis [1,12].

### 2.2. Effects on Skin, Bone, and Cartilage

Beta-ecdysone stimulated osteogenic differentiation of mesenchymal stem cells [27]. Bone differentiation markers like alkaline phosphatase (ALP) were induced in a dose-dependent manner, and transcripts of a major transcription factor of osteogenesis, Runx2, osteocalcin, and type I collagen were increased. This effect was prevented by an oestrogen receptor inhibitor, and a reporter gene indicated that 20E stimulated expression of this receptor. Symptoms of osteoporosis were alleviated in a mouse model of the disease, suggesting that ecdysterone can be useful in the treatment of osteoporosis.

Chronic oestrogen treatment bears several risks in postmenopausal conditions; therefore, it was of interest if 20E had a bone-protective effect but not an oestrogenic effect [28]. In ovariectomized rats, an animal model of postmenopausal condition, 20E increased bone mineral density in the tibia in a dose-dependent manner but did not bind to the oestrogen receptor in porcine uterine cytosolic extract. Serum CrossLaps, an adjunct of diagnosis, were lowered in both 20E and oestrogen treatment, but the osteocalcin, a marker of mineralization, decreased in oestrogen-treated animals and increased in 20E-fed animals. This indicated a non-oestrogenic protective effect of ecdysterone on bone formation. The joint, the epiphyseal cartilage, and the trabecular bone were also improved by ecdysterone in ovariectomized (OVX) rats [29]. This confirmed that ecdysterone itself or in extracts of plants (i.e., *Tinospora cordifolia*) might be protective in adiposity and elderly when joints are degrading faster.

The epidermal and dermal layers became thicker in 20E-treated rats than in control and β-oestradiol (E_2_)-treated OVX rats [30]. However, the thickness of the subcutaneous fat layer was intermediate in the 20E-treated group compared to the other two groups. The muscle thickness was larger in both 20E- and E_2_-treated rats. This suggested a change in the functionality of skin in response to 20E treatment.

Periodontal ligament cells (PLD) can be potentially used for tooth replacement. Ecdysterone increased proliferation and bone differentiation in these cells [31]. The bone morphogenic protein 2 (BMP-2) and ALP were increased in an extracellular-dependent kinase (Erk)/MAPK pathway-dependent manner. This highlighted 20E as a potential drug for periodontal therapy.

Another steroid hormone, glucocorticoid (GC), is used for chronic treatment, but it prevents age-dependent bone formation by influencing trabecular gain and cortical bone formation in young mice [32]. Moreover, 20E alone increased bone formation, and when added concurrently with GC, it compensated for the detrimental effect and prevented the GC-induced autophagy in bone marrow stromal cells, making it a promising neutralizer of GC side effects. The beneficial effect of 20E alone on bone formation was observed in both sexes [33].

Dexamethasone, a fluorinated glucocorticoid, is widely used in therapy mostly for its anti-inflammatory effect. Moreover, 20E inhibited NFκB activation similar to this drug; however, it has not been found to modulate the effect of dexamethasone in HeLa cells [34]. Seemingly in contradiction with the above report, 20E reverted UVB-increased expression of 11β-hydroxysteroid dehydrogenase type 1 (an enzyme converting cortisol to inactive cortisone, therefore preventing induction of GC receptor expression) and restored the level of GC receptor in HaCaT cells [35]. The counteraction with GC has been largely clarified by a study on hairless mice where 20E was found ameliorating the stress effect on the pituitary–hypothalamic–adrenal axis in UVB-induced photoaging [36]. It has reduced the aldosterone synthase and corticosterone levels in the adrenal gland and protected against the decrease in collagen level in the skin; therefore, it appeared a potential candidate to prevent skin aging. This again underlined the systemic nature of 20-hydroxyecdysone’s effect.

In vitro treatment with GC decreased osteoclast viability by decreasing differentiation markers and increasing a wide range of apoptotic factors [37]. This was reverted by 20E in agreement with the in vivo bone protective and GC side effect neutralizing potential of the phytoecdysteroid. Similar to mice, GC inhibited bone formation in rats by inducing apoptosis and inhibiting autophagy in lumbar vertebral tissue [38]. This effect was attenuated by ecdysterone with the inhibition of apoptosis and stimulating autophagy, strengthening 20E as a promising candidate for treatment of osteoporosis.

Interleukin-1β (IL-1β) is inducing osteoarthritis (OA) produced by an imbalance between the catabolic and anabolic activity of chondrocytes. The catabolic process is regulated by the hypoxia-inducible factor 2 alpha (HIF-2α) and elevated MMP3 production in chondrocytes and synovial cells. The anabolic process is marked by collagen type II (Col2a1) gene expression. In addition, 20E scavenged the increase in catabolic processes and decrease in anabolic processes and largely restored the balance in primary explanted articular chondrocytes [39], confirming a protective effect in OA.

Bone marrow stem cells (BMSC) might be important in helping ontogenesis. In addition, 20E increased osteogenic markers in these cells when differentiated and ameliorated experimentally induced osteonecrosis in the rat model by enlarging femoral head tissue size. Parallel to the osteogenic transcription factor RUNX2, collagen chain COLIA1, osteocalcin, the IP3-kinase, and Akt phosphorylation were also increased in the in vitro and in vivo systems [40]. This suggests 20E as a therapeutic agent in osteogenic diseases.

Osteoarthritis can be induced by the injection of collagenase in the mouse knee [41]. The symptoms were alleviated by 20E supplementation. The effect involved amelioration of pro-inflammatory cytokines, rescuing FOXO1 protein expression in the nucleus that inhibited transcription and translation of members of the ADAMTS protease group contributing to degrading extracellular elements. Others reported that ecdysterone stimulated osteogenic osteoblast proliferation and differentiation in vitro and bone regeneration in vivo [42]. However, this happened via the BMP-2/Smad/Runx2/Osterix pathway, which revealed a new connection as BMP-2 had been related earlier to the Erk/MAP kinase pathway [43]. This indicated a novel therapy target for an osteonecrosis cure.

### 2.3. Influence on Nervous System

Ecdysterone could attenuate vasospasm and alleviate neurological deficits that happen as a consequence of subarachnoid haemorrhage in rabbits [44]. It was hypothesized that it can also protect against oxidative cellular damage. The PC12 cell line is an in vitro model of neural differentiation after various brain traumas. Moreover, 20E helped the recovery of PC12 cells after CoCl_2_-induced injury, including reduction of reactive oxygen species, a decrease in depolarization of the mitochondrial membrane, and the release of cytochrome C from mitochondria. In line with this, the Bcl2/Bax ratio was elevated, and caspase 3 activity was abolished. Therefore, 20E was an anti-oxidant and acted against the mitochondrial apoptotic pathway, suggesting that it can be used to prevent hypoxic-ischaemic brain damage in stroke [45]. Similarly, in cerebral focal ischaemia of rat 20E, increased angiogenesis and astrocyte activity paralleled with increased microvascularization and formation of more and longer nerve endings [46]. The anti-oxidant effect of 20E was also investigated in rat brain and B35 cells [47]. After occlusion of the cerebral artery, 20E decreased the infarct volume and the neural deficit score; furthermore, it restored anti-oxidant capacity, decreased malondialdehyde level, and decreased the number of TUNEL-positive cells and the number of cells with cleaved caspase 3 in the cerebral cortex. Hydrogen peroxide treatment of B35 cells induced damage and oxidative stress. Ecdysterone markedly attenuated ROS/RNS production, dissipation of mitochondrial membrane potential, descent of anti-oxidant potential, level of malondialdehyde, and level of intracellular Ca^2+^ levels. It also reduced iNOS expression by inhibiting NFκB activation and inhibited activation of the ASK1-MKK4/7-JNK stress signalling pathway. Together these indicate that 20E was protective against ischaemic injury and oxidative stress in neural tissue.

Oxidative damage in the hippocampus CA1 area causing significant memory loss also happens in experimentally induced diabetes type I as a side effect [48]. In the molecular background, the NFκB level is increased while the SOD, catalase, glutathione peroxidase (GTHpx), reductase (GR), and BDNF declined. Interestingly, 20E, especially at higher concentrations, reverted these changes, attenuating memory loss.

The monoaminergic system is related to memory loss and decline in cognitive function in neurological symptoms as Parkinsons and Alzheimers diseases [49]. In a mouse model of Parkinson disease, 20E protected dopaminergic neurones, mitigating mitochondria-mediated apoptosis and inducing anti-oxidant enzymes (SOD, catalase, GTHpx, and GR). It also increased the Bcl2/Bax ratio and decreased cytochrome C release and caspase activities with increased activity of the Nrf2 (phosphoinositide-3-kinase-nuclear factor E2-related factor 2) pathway. This showed the 20E protected against neurotoxicity by its anti-apoptotic and anti-oxidant capacity and may have a potential in the therapy of Parkinson disease [50]. In addition, 20E was also protective against oxidative stress and apoptosis in PC12 cells treated with MPTP, an inductor used in animal models of neurological disease like Parkinson [51]. Akt signalling and nuclear translocation of Nrf2 and HO-1 (hemoxigenase-1) expression were elevated as a part of the anti-oxidant response. However, the activity of NF-κB and calpain was not affected.

Glutamate toxicity has been found in the pathology of many neurological diseases. Ecdysterones derived from *Rhaponticum chartamides* were also preventive against glutamate-induced excitotoxicity in rat brains [52]. The protective mechanism appeared via a similar mechanism as follows: the upregulation of PI3K, Akt, and mTOR and the downregulation of the apoptotic enzyme cleaved caspase-3 and GRIN2B, a glutamate NMDA receptor involved in rare neural development disorders. Stress, anxiety, and depression were reduced in mice by a 20E-enriched fraction of *Pfafia glomerulata* roots [53]. The anti-oxidant enzymes were activated, and the oxidative markers were reduced. The concentration of NO increased in the striatum, which may improve memory function and anti-oxidant activity. High-intensity interval training (HIIT) can be a strategy in the treatment of cognitive decline and oxidative stress. Both of these are common symptoms in Alzheimers disease (AD), characterized by the deposition of beta-amyloid protein (Aβ). In the Aβ-induced rat AD model, spatial avoidance learning and memory were deprived. These functions declined parallel with anti-oxidant enzymes (SOD, CAT, and GTx). HIIT alleviated learning and memory loss together with the anti-oxidant potential in the animals; however, in combination with 20E, it was more effective for neuronal protection [54].

Smooth muscle contraction and relaxation depend on the release of neurotransmitters such as acetylcholine (ACh). However, 20E enhanced ACh release, inducing contraction of isolated gastric smooth muscle preparation, while the extract of the ecdysterone-rich plant, *Rhaponticum carthamoides,* presented a significant inhibitory effect on contractile properties. Considering also the plausible differences in the applied dose of 20E, this might have exemplified that ecdysterone-containing plant extracts do not always exert the same effect as the effective compound in pure form [55].

### 2.4. Actions on Inflammation and Apoptosis

Furthermore, 20E has also been reported to have an anti-inflammatory and anti-apoptotic effect by preventing interleukin-1β-caused injury in rat chondrocytes [56]. The Bax expression and p53 phosphorylation were inhibited, and the Bcl-xl effect was promoted in these cells. Simultaneously, 20E decreased Caspase-3 activity; prevented matrix degradation by downregulating MMP 3, MMP 9, and cyclooxygenase expression; and inhibited NF-κB p65 phosphorylation. This effect was similar to the mechanism of several CRMs, particularly that of curcumin, aspirin, and resveratrol [57,58].

The anti-apoptotic effect of 20E was also observed in the human neuroblastoma cell line [59]. A neurotoxin, 6-hydroxidopamine (6OHDA), was used to induce apoptosis in a model of detrimental loss of dopaminergic neurones in Parkinson disease. In addition, 20E protected against apoptosis in a mitochondria-dependent manner; it downregulated Bax and PUMA (p53-upregulated modulator of apoptosis), suppressed the loss of mitochondrial membrane potential (ΔΨm), and attenuated cytochrome C release and caspase 9 activity. It also inhibited the p38MAPK-dependent promoter activity of p53 that contributed to cell protection. ShRNA inhibition of apoptosis silencing-regulating kinase (ASK1) and blockade of reactive oxygen species (ROS) prevented the protection, indicating a mechanism for the anti-apoptotic effect. In addition, 20E alleviated collagen-induced rheumatoid arthritis in rats [60]. The treatment decreased paw swelling, arthritis score, and thymus spleen index; the level of articular elastase; and anti-collagen IgG. Biochemical parameters like anti-oxidants (superoxide dismutase, catalase, and glutathione) and inflammatory markers (NO, IL-1β, IL-6, TNF-α, and NFκB p63) were downregulated. Therefore, ecdysterone may effectively eradicate the inflammatory cascade and oxidative stress in rheumatoid arthritis of synovial joints.

Radiation-induced oral mucositis (RIOM) is rate limiting in the treatment of head, neck, and other cancers. Ecdysterone improved healing of the mucosa by augmenting activity of the Ras-Raf-Erk pathway and increasing the proliferation of matrix cells [61]. In addition, 20E was even more ameliorating in combination with paeonol (a compound derived from Cortex Moutan, the root bark of *Paeonia suffruticosa* Andr.), and calculational chemistry showed that ecdysterone-paeonol may interact with 19 targets that are functional in RIOM, including apoptosis, inflammation, proliferation, and wound healing [62]. In the early stages of RIOM, the same research group [63] also found that 20E treatment attenuated the radiation-induced decrease in cellular superoxide dismutase and the increase in malondialdehyde concentration. It also upregulated anti-apoptotic Bcl2 and downregulated pro-apoptotic Bax and the activated caspase 3. These supported remarkable anti-apoptotic and anti-oxidant properties in the early phase of irradiation.

Curiously enough, 20E selectively decreased viability in a triple receptor-negative breast cancer cell line in contrast to other breast cancer cells and non-cancerous controls [64]. This effect was manifested by pro-apoptotic activity altering PARP, Bax, Bcl-2, and caspase 3 activity and induction of autophagy-associated proteins. The differential effect was suggested to be associated with altered molecular levels, including receptors, biological status, and genetic properties, which are all plastic in behaviour in tumour.

### 2.5. Impact on Liver and Adipose Tissue

The liver is the centre of metabolism and shows altered functions in diabetes mellitus (DM) as it secretes glucose into the blood plasma instead of storing it in hepatocytes. This happens because the diabetic liver is not sensitive enough to insulin and responds more to glucagon; thereby, gluconeogenesis and glycogenolysis dominate over glycolysis and glycogenesis. When the liver keeps secreting glucose into the blood plasma, it makes hyperglycaemia. Reverting this effect, 20E decreased the rate-limiting enzymes of gluconeogenesis like phosphoenolpyruvate carboxykinase (PEPKC) and glucose-6-phosphatase (G6Pase) in vitro in hepatocytes and in C56BL/6 mice kept on a high-fat diet (HF). It also elevated adiponectin, known to reduce gluconeogenesis and enhance glycolysis and fatty acid oxidation in the liver. The daily oral administration of 20E decreased body weight, hyperglycaemia, and plasma insulin level and ameliorated insulin resistance and obesity in the treated compared to untreated HF control mice [65].

The effect of 20E has also been studied in the streptozotocin-induced type I diabetic model in rats [66], known to decrease body weight. After 30 days of oral administration, the plasma levels of glucose, glycated haemoglobin (HbA1C), and insulin were lowered. The level of glucose uptake enzyme (hexokinase) and the key enzyme of hexose monophosphate shunt/glucose direct oxidation (glucose-6-phosphate dehydrogenase) were increased, whereas gluconeogenic enzymes like glucose-6-phosphatase and fructose-1,6-bisphosphatase levels were decreased. The effect of 20E was comparable to the antidiabetic drug glibenclamide, known to promote insulin secretion of beta cells.

Diabetes mellitus is often associated with the complication of hyperlipidaemia. This represents an additional risk for cardiovascular functions. Oral administration of 5 mg 20E/kg body weight per day to STZ-induced (type I) diabetic rats for 30 days ameliorated the unfavourable changes of lipid parameters [67]. Namely, it reduced the fasting blood levels of glucose, cholesterol, free fatty acids, glycerol, phospholipids, low-density lipoproteins, very low-density lipoproteins, and 3-hydroxy-3-methylglutaryl CoA reductase in the liver and kidney and elevated the high-density lipoprotein, lipoprotein lipase, and lecithin cholesterol acyl transferase in the plasma compared with diabetic control rats. This effect of 20E was like that of the antidiabetic drug glibenclamide and can be explained by the improved insulin level [58,67].

Desert gerbil (*Gerbillus gerbillus*) represents a natural model of human metabolic disorder and non-alcoholic fatty liver disease [68]. In STZ-induced diabetes of this rodent, the insulin level was increased, and the plasma glucose level was decreased by short-term i.p. injection of 5 mg/kg body weight of 20E. The glycogen store was also increased, lipid peroxidation was reduced, and metabolic disorders were counteracted by 20E. Interestingly, 20E administered only once, 3 h before animal sacrificing, was enough to ameliorate plasma levels of insulin and glucose and moderate degranulation of beta cells of pancreatic islets of Langerhans [69]. Another gerbil species (*Gerbillus tarabuli*) showed histopathological alteration and metabolic problems after long-term consumption of a high-carbohydrate diet (HCD). Moreover, 20E reduced the pathological changes in a dose-dependent manner. In addition, a hepatoprotective effect was demonstrated by decreased levels of ALT, AST, and hepatic malondialdehyde in blood plasma [70]. Considering the high efficacy in gerbil, the prevalence of 20E in blood plasma has been studied after per os or i.p. administrations [71]. This parameter, called bioavailability, is measured by the area under the curve (AUC) on a graph showing plasma concentration in time after administration. The single administration of 50 mg 20E/kg bw. by oral gavage in gerbil revealed 12% bioavailability of 20E, about 10-fold higher than after a similar administration in rats and humans (1–2%). However, a 10-fold lower dose, the i.p. injection of 5 mg/kg bw, did not result in such a big difference in gerbil compared to rats or humans. The high prevalence of 20E in the digestive and nutritive system in case of a higher dose makes gerbil an interesting object to study.

In addition, 20E appears to counteract fat absorption and diet-induced obesity [72]. This was first observed in connection with the quinoa extract (Q) enriched in the moulting hormone. The Q or the similar amount of pure 20E decreased adipocyte development in mice when supplemented to the high-fat diet (HF) without modifying weight gain. This was paralleled with a decline in mRNA levels of genes involved in fat storage compared to HF mice. The glucose tolerance or metabolism was not affected. Interestingly, Q + HF treatment displayed lower mRNA levels of inflammation and insulin resistance markers and reverted the HF-diet-elevated mRNA levels of the mitochondrial uncoupling proteins in skeletal muscle. The optimized Q extract significantly lowered fasting blood glucose in obese, hyperglycaemic mice [73]. The expression of PEPCK was also downregulated by Q in accordance with a previous report [65]. This gluconeogenic enzyme promotes triglyceride synthesis in adipocytes. It was also found that Q counteracted the HF-related elevation of lipoprotein lipase (LPL) and peroxisome proliferator-activating receptor-γ mRNA levels, indicating a decrease in fat-storing capacity in adipose tissues. The faecal lipid ratio was also increased without altering the stool amount. This supported the usage of quinoa extract and 20E as an anti-obesity supplement in the diet. The above observations had been confirmed by metabolic energetics [74]. Both Q and 20E increased global energy expenditure calculated from the ratio of exhaled carbon dioxide and oxygen consumption when supplemented to an HF diet in mice. Because of the lower C/O ratio in lipids compared to the 1/1 in glucose, the near one ratio of VCO_2_/VO_2_ (respiratory quotient) indicates more glucose oxidation, while lower than one (0.7–0.8) shows increased lipid utilization. This happens because lipid oxidation consumes more oxygen as compared with carbohydrates when referred to one carbon atom. In addition, in triglyceride catabolism, the enzyme pyruvate dehydrogenase producing CO_2_ is mostly out of use because the glycerol-derived pyruvate is utilized to produce oxaloacetate by the pyruvate carboxylase reaction (using CO_2_). The oxaloacetate then helps the TCA cycle to accept acetyl-CoA coming from beta oxidation of fatty acids during lipid breakdown. Following this study, the quinoa extract was suggested for alleviation of non-alcoholic fatty liver disease [75]. Similarly, the extract of the *Ajuva iva* plant was found to significantly ameliorate alloxan-induced diabetes in rats by lowering blood glucose level; improving insulin and protein levels; and reducing blood urea nitrogen, creatinine, triglyceride, cholesterol, and lipid peroxidation. Alloxan kills pancreatic β-cells (inducing type I diabetes), but the Ajuga extract, rich in ecdysteroids, promoted regeneration from this detrimental effect [76].

### 2.6. Calorie Restriction Mimetics and 20E

Additionally, 20E appeared to decrease body weight and adiposity and improve lipid parameters similar to calorie restriction (CR). CR is a method of deliberate reduction in food intake with 10–50% with careful avoidance of malnutrition, that is, providing a sufficient amount of macro- and microelements, vitamins, and dietary fibres [77]. It has an established beneficial effect on health and life span in laboratory animal models from yeast to rhesus monkeys [78,79]. It also appears to be a promising method to improve health conditions and extend healthy life in humans [80]. A two-year-long clinical trial with CR called CALERIE (Comprehensive Assessment of Long Term Effects of Reducing Caloric Intake) led to a straightforward conclusion about the anti-aging effects, including weight loss, improvement in cardiovascular risk parameters, and an increase in insulin sensitivity in healthy non-obese adults [81]. It was also recognized that diet composition (the protein/carbohydrate ratio) and adherence to diet restrictions influenced the risk parameters [82,83]. The age-related DNA methylation pattern in long-term calorie restrictions [84] and the telomere attrition were also interestingly complicating the scene about the potential effect [85]. However, the long-term outcomes of CR still remain to be explored for the role in improving human health, protecting against age-related ailments and diseases, and extending healthy lifespan [86].

It has been recognized that certain metabolic pathways and signal transductions are involved in the mechanism of CR action [87]. Pharmacological compounds mimicking calorie restriction (CRM) became widely used in the medication of metabolic diseases as a substitute for decreased calorie intake [88]. Although the mechanisms of action of CRMs are diverse and not always properly revealed yet, it can be stated that these typically achieve weight loss, change metabolic rates, prevent accumulation of reactive oxygen species, and influence pathways that are also activated in calorie restriction. These pathways are mostly, but not entirely, the insulin/insulin-like growth factor, the target of rapamycin (TOR), the adenosine monophosphate-activated protein kinase (AMPK), and the Sirtuin signalling [78]. There are more than 20 compounds with reported CRM effects (reviewed in [58]), among them are, for example, the metformin used for more than 60 years to ameliorate diabetes. Here belongs resveratrol, the naturally occurring polyphenol, 2-deoxyglucose, and the inhibitor of glycolysis, nicotinamide mononucleotide (NMN) and nicotinamide riboside (NMR), which are constituents of many foods like pork or vegetables and curcumin. A compound of oriental spice, hydroxyl citric acid (HCA), an inhibitor of ATP-citrate lyase found in leaves of Garcinia species in the South Asian region, alpha-ketoglutarate, a key factor and regulator of the TCA cycle, and aspirin, an analgesic, antipyretic, and non-steroidal anti-inflammatory drug that inhibits the cyclooxygenase pathway, are all considered as CRM. Although the CRMs target different pathways and are used to ameliorate a variety of pathological conditions, they are all assumed to have anti-aging effect [58]. Although it is debated if they indeed extend the entire life span, the anti-aging benefit is recognized as the elongation of the period spent in good health, “healthspan”, like in the case of metformin [89].

**Figure 2:**
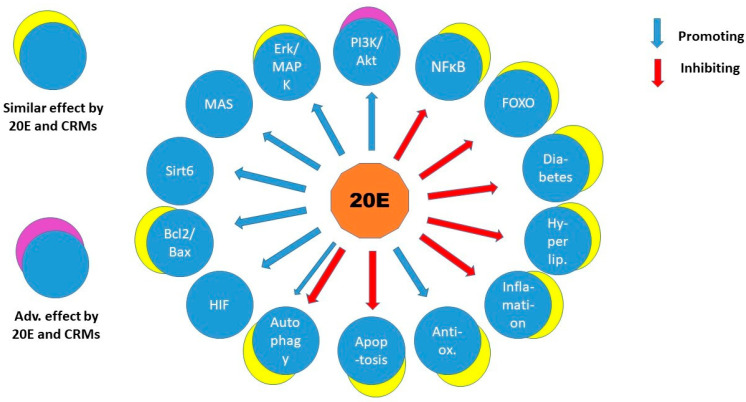
The similarity of effects of 20-hydroxiecdysone and CRMs on signalling molecules and cellular and metabolic processes. The overlapping circles indicate similar or (in one case) adverse effects. Hyperlip.—hyperlipidaemia, other explanations are present in the text. Note that the adverse effect was exerted on PI3K/Akt, a major pathway promoted by 20E in the anabolic effect, because it is inhibited by compounds mimicking calorie restriction.

Although most of the mechanisms of CRM actions are still subjects of investigations, it can be noted that they influence similar cellular pathways and pathological conditions as 20-hydroxyecdysone does (Figure 2). One can be curious if 20E might be a CRM in mammals. Indeed, quite a few examples have been reported about influence on enzymes, pathways that are also activated in CR. Ovariectomized rats fed with a thigh-fat, high-fructose diet (OHFFD) are animal models of “metabolic dysfunction-associated fatty liver disease” (MAFLD), a non-alcoholic fatty liver disease. Dietary supplementation of 20E in OHFFD increased the phosphorylation of AMPK and acetyl-CoA carboxylase while reducing the expression of fatty acid synthase in liver and adipose tissue. Moreover, 20E also increased expression of carnitine palmitoyltransferase-1 in the liver and reduced expression of sterol regulatory element-binding protein-1 in adipose tissue. The above alterations decreased fatty acid biosynthesis and lipogenesis and increased lipolysis/beta-oxidation; therefore, 20E ameliorated hepatic steatosis combined with overweight, diabetes, or other metabolic risk factors, and this was achieved without altering calorie intake in OHFFD rats [90]. This effect was similar to that of Pioglitazone (PIO), a peroxisome proliferator-activated receptor-gamma (PPAR-γ) agonist. Not incidentally, PIO is a CRM to ameliorate steatosis; however, with unwanted side effects [91].

Sirtuins, one of the main targets of CRMs, have been reported only once in interaction with 20E. Endothelin cells grown in 3-D culture (HUVECs) had been treated with TNF-α, which induced CD40 expression via NF-κB activity. This mechanism starts inflammation because CD40 is inducing IF-β, an inflammatory factor. NF-κB p65 acts in an acetylated form but becomes deacetylated by Sirt6, a nucleus localized protein, therefore decreasing its activity. In docking experiments, 20E binds to Sirt6 and stimulates its expression and also stabilizes it in HUVECs. This scenario protects against endothelial inflammation [13] and is similar to the anti-inflammatory feature exerted by some CRMs, like metformin, curcumin, and alpha-ketoglutarate [58].

One of the most resembling effects of 20E on calorie restriction has been reported in C57BL/6J mice fed a high-fat diet (HF). Body weight and fat tissue mass have been decreased by 20E compared to only HF control. Remarkably, the hepatic expression of key gluconeogenic enzymes (PEPCK and G6Pase) has also been decreased, ameliorating the pathogenic role of the liver in diabetes of HF mice [65].

## 3. Discussion

Ecdysterone has a fairly similar anabolic influence in vitro on various cell types and in vivo in animal models and clinical trials. This effect well conforms with the anti-oxidant, anti-hyperglycaemic; anti-obesity; anti-apoptotic; and hepato-, neuro-, immuno-, osteo-, chondro-, and other protective properties. The impact is more apparent in various pathological models than in healthy conditions. This fits well with the adaptive role of 20E in helping to restore somatic imbalance. In view of the distinct functions of tissues and organs within the organism, it must be taken into consideration that finding a primary target is difficult when looking at systemic outcomes. The research on the effects of 20E has made progress in many areas, and it seems to approach an array of targets instead of a major one. Namely, the cooperation of the oestrogen receptor beta (ERβ) in a membrane-attached palmitoylated form with the MAS receptor in C2C12 myogenic cells looked feasible to interpret the pleiotropic effect of the hormone. Others proposed that 20E stimulated Sirt6 activity in TNF-α-induced HUVEC cells, and Sirt6, as a nuclear protein, deacylated the transcription factor NFκB; therefore, CD40 was not induced, and this prevented inflammation. According to in vitro assays, 20E could bind to Sirt6 directly and stimulate its activity, suggesting a starting point for this mechanism.

In silico analysis, although indirectly, may still reflect several possible candidates of ecdysterone effect. Searching for pharmacological targets of ecdysterone-rich Cyanotis arachnoidea extract revealed a network of genes [92] similar to the in vitro and in vivo experiments overviewed here (Figure 2). In addition, 20E also can exert in vitro inhibition on activities of pharmacologically important enzymes like acetylcholinesterase, butirylcholinesterase, tyrosinase, and α-amylase not in disagreement with the complexity of the hormone action [93]. Special methods can optimize the screening and extraction efficacy of ecdysteroids in order to improve the pharmaceutical treatment of pathological conditions [94]. These types of approaches are also used to highlight the anti-inflammatory and enzyme-inhibitory properties of the hormone and other compounds in folklore herbs [95].

Most recently, the upregulation of energy production has been put forward as the main reason for 20E’s anabolic activity in myoblasts (C2C12) and embryonic fibroblasts (NIH3T3) [96]. The authors, just like other researchers, found that the PI3K/Akt/mTor is the major signalling pathway involved in increased protein synthesis and anti-oxidant effect, accompanied by increased C1 metabolism as newly reported data. However, this hypothesis is still not elucidated better, that is, how would 20E act directly on glucose uptake from plasma (according to its anti-hyperglycaemic activity)? If the entire aerobic and anaerobic glucose degradation is named as the mediator, it is worth appreciating that it includes highly regulated alternative pathways. An anabolic process always needs energy, but boosted energy production is not necessarily contributing to anabolism. Intermediates of the TCA cycle can be used for anabolic processes but then are less available to provide reduced cofactors for oxidative phosphorylation. Key enzymes, feedback inhibitions, and cofactor availability make the entire glucose degradation not so straightforward as it may look. For example, Shuvalov et al. [96] reported that 20E elevated LDH expression together with increased terminal oxidation. This suggests both lactate and acetyl-CoA (AcCoA) production from pyruvate, the end product of aerobic glycolysis. AcCoA must enter the citric acid cycle (CAC) to feed oxidative phosphorylation. However, when AcCoA cannot enter CAC, it acts with negative feedback on its source enzyme, the pyruvate dehydrogenase. When CAC is inhibited (by excess ATP and reduced coenzymes), citrate leaves the mitochondria and feeds fatty acid synthesis (FAS) with AcCoA. Therefore, CAC and FAS are two competing pathways that need delicate regulation. FAS is a major anabolic process and is required for phospholipid synthesis, including cardiolipin, an essential component of the inner mitochondrial membrane. In line with this, a multivariate analysis of the metabolome has recently found that 20E supplementation increased phosphatidylcholine, a major phospholipid in the serum of post-trained athletes [97]. Citrate after leaving the mitochondrion is converted to AcCoA and oxaloacetate by the ATP citrate lyase (ACL). This enzyme is also required for muscle growth as it increases cardiolipin for mitochondria. Muscle hypertrophy or alleviation of atrophy depends on the IGF-I/ACL/cardiolipin pathway [98]. ACL limits AcCoA availability, therefore, regulating histone acetylation at MyoD acting sites of chromatin needed for muscle differentiation [99]. Recently, ACL of skeletal muscle has been found to increase in a special group of heart patients who responded to resistant exercise with muscle hypertrophy and ameliorated cardiac pathology only when they received a calorie restriction diet [100]. This observation highlights the paradoxical effect of calorie restriction, that is, the enhancement of anabolic processes when energy is needed [101]. The above scenario with the central role of ACL does not refute Shuvalov et al.’s suggestion [96], but it may imply that 20E acts similarly to calorie restriction. Recently, it has been reported that starving cells can overcome the difficulty of balancing catabolic and anabolic processes using the fusion and fission cycle to sequester a subset of mitochondria specialized on synthetic processes instead of oxidative phosphorylation [102]. Future studies may enlighten whether 20E has similar potential. Besides that, it is worth keeping in mind that the different tissues can be orchestrated under 20E influence. In liver hepatocytes, 20E inhibits FAS in vivo; meanwhile, it promotes fatty acid degradation [90], showing the opposite response to that of myoblasts and fibroblasts in vitro [96]. Therefore, different tissues can react differently to 20E, mostly according to their functions within the body. Metabolism consists of interlinked catabolic and anabolic processes; it seems that 20E affects both kinds but influences tissues depending on the need of the entire organism. That is why this secondary plant product is often also called adaptogenic, next to anabolic [103,104,105].

Since 20E appears to act on both mitochondrial function [59,106] and fat metabolism [67,70,74,75,90], it is an intriguing question if it can influence fat browning. This dynamic adaptation of adipose tissue forms beige or brite (brown in white) fat cells. Brite fat behaves as an intermediate compared to white and brown adipose tissue; it produces heat from glucose uptake and contributes to body temperature in non-shivering thermogenesis. This is in contrast with white fat cells, which synthetize triglyceride from glucose and fatty acids. Besides metabolic consequences, such transformation also has an endocrine impact [107]. A study has been reported in gerbil on a high-fat diet that 20E supplementation increased glycogen storage instead of lipid droplets in the interscapular brown adipose tissue compared to the controls [108]. Although the significance of glycogen storage in brown adipose tissue is still not fully interpreted, it shows that the effect of 20E is not neutral on this tissue.

When looking at beta-ecdysterone’s effect on animal models, hardly any detrimental effect can be found. The latest report has been about the genotoxicity of the 0.0823 mg/kg lowest effective dose on rat bone marrow in 2016 [109]. This amount is around the recommended one that can be used for daily human consumption. Considering the health relevance of this information, it is of concern that no follow-up of this study has appeared in the last eight years in the literature.

20E appears protective against osteoporosis [27,38], therefore acting against aging. In metabolic diseases, it is particularly noticeable that it has a lot of similarities with calorie restriction. It decreases weight without altering diet and is anti-hyperglycaemic, anti-lipidemic, anti-oxidant, anti-apoptotic, and anti-autophagic. Calorie restriction mimics appear to influence the same signalling pathways and body conditions in a similar way as 20E does. However, there are exceptions, like the PI3K/Akt/mTor in muscle. The activity of IGF-1/IGFR that elevates PI3K/Akt/mTor has been shown to act against longevity in animal [110] and human studies [111,112]. However, it must be kept in mind that 20E is anabolic, and anabolism is important for muscle gain. Maintaining muscle mass and mobility is a prerequisite of a long and healthy life; therefore, this part of the ecdysterone effect may also be useful for achieving longevity.

## 4. Conclusions

20E influences multiple cellular pathways and tissues and has at least three suggested molecular targets in its action; however, none of these has been proven to connect directly in vivo. Two of the proposed molecular targets have been studied in myoblasts, 1 in endothelial cells, and the increase in energy boost in myoblasts and fibroblasts has been proposed as a major cause for the anabolic effect. This already implies that 20E has a systemic effect that probably cannot be attributed to one molecular target, although the Sirt6 stimulation in endothelial cells looks promising for an explanation of pleiotropy. Although 20E is not considered a CRM, it similarly influences a number of cellular processes and pathological conditions to calorie restriction mimetics. Nonetheless, PI3K/Akt/mTor, the major pathway that appears to drive 20E’s anabolic effect, is adversely affected by most CRMs. Nevertheless, the comparison of 20E and CRMs seems interesting for further research of mechanisms and potential in anti-aging. As more studies will be performed on the 20E effect, the knowledge of the mechanism of action is likely to broaden significantly. It seems that approaches involving network analysis of molecular interactions and pathways may bring an inspiring view of this field of research.

## Data Availability

Not applicable.
